# The impact of widowhood on household consumption among middle-aged and older adults: evidence from China

**DOI:** 10.3389/fpubh.2025.1635832

**Published:** 2025-09-01

**Authors:** Xianfu Jiao, Lijin Peng, Xu Si, Xujin Yang

**Affiliations:** ^1^School of Insurance, Central University of Finance and Economics, Beijing, China; ^2^Business and Tourism School, Sichuan Agricultural University, Yaan, China; ^3^School of Finance, Hunan University of Finance and Economics, Changsha, China; ^4^School of Mathematics and Statistics, Hunan University of Technology and Business, Changsha, China

**Keywords:** widowhood, household consumption, saving, income, psychological consumption

## Abstract

**Background:**

As China enters a phase of accelerated population aging, the number of individuals aged 65 and above had surpassed 217 million by the end of 2023, accounting for approximately 15.38% of the total population. This demographic shift has triggered an unprecedented wave of social challenges. While birth, aging, illness, and death are the natural parts of human life, widowhood functions as an “invisible threat” to the well-being of middle-aged and older adults. Accordingly, this paper investigates the impact of widowhood on household consumption among middle-aged and older adults, along with the underlying mechanisms.

**Methods:**

This paper utilizes data from the China Health and Retirement Longitudinal Study (CHARLS) from 2011 to 2020, and applies a difference-in-differences (DID) approach to assess the impact of widowhood on household consumption among middle-aged and older adults. Heterogeneity analysis is conducted based on household head characteristics and consumption structure to explore the differential impacts of widowhood. Moreover, the mechanisms are analyzed through three mechanisms: income, psychological consumption, and household saving rate.

**Results:**

Widowhood leads to a reduction in household consumption among middle-aged and older adults. However, the magnitude of this decline is relatively modest. Heterogeneity analysis reveals that widowhood exerts a significantly adverse impact on rural households, whereas no significant effect is observed among urban households. The negative effect on consumption is more pronounced among middle-aged individuals than among older adults. The reduction in medical expenditures caused by widowhood is greater than that in non-medical consumption. Mechanism analysis indicates that widowhood directly reduces household consumption by lowering income and increasing psychological consumption, while indirectly reducing consumption by raising the household saving rate.

**Conclusion:**

Widowhood negatively affects household consumption among middle-aged and older adults, primarily due to reduced income. It is therefore recommended to enhance the social security and psychological support systems, strengthen income and healthcare protection for rural middle-aged and older adults in a context-sensitive manner, and promote scientific household saving strategies to mitigate widowhood-related risks.

## 1 Introduction

As China enters a phase of accelerated population aging, the number of individuals aged 65 and above had surpassed 217 million by the end of 2023, accounting for approximately 15.38% of the total population^1^. This demographic shift has triggered an unprecedented wave of social challenges. This massive silver-haired population not only places extraordinary demands on pension systems and healthcare resources, but also imposes unprecedented pressure on various public service infrastructures. At this historical juncture, improving the quality of life for middle-aged and older adults is not only an essential step in mitigating the impact of population aging, but also a fundamental strategy for ensuring sustainable social development. While birth, aging, illness, and death are the natural parts of human life, widowhood functions as an “invisible” threat to the well-being of middle-aged and older individuals. If timely emotional and psychological interventions are not implemented to prevent the deteriorating effects of widowhood on the health of middle-aged and older adults, the consequences may ripple outward, compromising broader social welfare and economic vitality. Accordingly, the question of how to establish an integrated support system—through policy innovation and coordinated social services—that can both alleviate grief and facilitate recovery has become a central imperative in responding to the challenges of population aging and safeguarding the nation’s long-term development.

The trauma of widowhood is not merely psychological; it constitutes a comprehensive shock to the lives of older adults. In addition to increased risks of depression and social isolation, widowed individuals often experience a rapid decline in self-care capacity and a substantial deterioration in overall quality of life (1, 2). Even in developed countries with relatively robust pension systems, the death of a spouse can lead to the collapse of previously stable household finances, forcing many widowed older adults into poverty (3–6). This tragedy is equally prevalent in developing countries (7). Income loss frequently results in reduced consumption and prompts households to increase precautionary savings (8). Moreover, a single health crisis can significantly reshape household investment preferences and shift the boundaries of risk tolerance (9). Meanwhile, end-of-life medical expenses often impose a substantial financial burden on families (10).

In fact, a substantial body of bereavement research has focused on the health consequences of loss. Widowhood not only increases the risk of depression among older adults but also raises the likelihood of functional disability (2). In terms of intergenerational effects, the death of a parent has been shown to have significant adverse impacts on the mental health of children (11–13). Boys who lose their fathers tend to exhibit poorer mental health outcomes, while girls who lose their mothers are more prone to developing depression (14) and engaging in self-injurious behaviors and other harmful health-related activities (15). Moreover, the death of a child significantly increases the risk of premature mortality among bereaved parents (16). A higher number of bereavement experiences is also correlated with elevated activation of depression-related genes (17).

However, existing research exhibits two primary limitations. On the one hand, most studies focus on the psychological and physical health outcomes of widowhood, or on emotional well-being more broadly (2, 11–14), while offering limited direct analysis of changes in household consumption patterns. On the other hand, even studies that address the economic consequences of widowhood tend to concentrate on indicators such as income, asset holdings, and poverty rates (3–6), with relatively little attention paid to the total volume of household consumption or the structural composition of expenditures.

In light of these limitations, this paper utilizes data from the CHARLS spanning from 2011 to 2020 and applies a DID approach to examine the impact of widowhood on household consumption among middle-aged and older adults, as well as the underlying mechanisms through which these effects operate. This paper makes the following potential marginal contributions:

First, it shifts the analytical perspective from health outcomes to consumption behavior by systematically evaluating how widowhood affects household consumption patterns, thereby contributing new empirical evidence from China on the behavioral consequences of bereavement among older populations. Second, drawing upon longitudinal CHARLS panel data from 2011 to 2020 and implementing a DID framework, the study identifies causal effects of widowhood on consumption outcomes. The empirical findings indicate that widowhood significantly reduces household consumption. This adverse effect is particularly pronounced in rural households, while no statistically significant effect is observed in urban settings. Furthermore, the negative impact is greater among middle-aged individuals than among older adults. Notably, the reduction in medical consumption is more substantial than that in non-medical consumption. Third, this paper develops a framework in which widowhood affects household consumption through three mechanisms: a decline in household income, an increase in psychological or emotional spending (referring to expenditures aimed at emotional comfort), and a rise in the household saving rate. From the perspectives of both direct and indirect effects, the analysis empirically examines the mechanisms through which widowhood influences consumption. The results show that widowhood directly reduces household consumption by decreasing income and increasing psychological expenditures, while indirectly reducing consumption by encouraging higher savings behavior.

The structure of this paper is as follows. The second section introduces the methodology; the third section presents the results; the fourth section offers further discussion; and the fifth section concludes with a summary and policy recommendations.

## 2 Methods

### 2.1 Research hypothesis

Widowhood causes serious psychological trauma and financial disruption for middle-aged and older adults (1, 2). More critically, it may even lead surviving spouses to fall back into poverty (3–6). As household income declines, risk tolerance and investment preferences may also shift accordingly (9). In addition, families may further reduce consumption and engage in precautionary saving behavior (8). However, according to the life-cycle hypothesis, individuals aim to maximize utility by smoothing consumption over time. Therefore, while widowhood may lead to a reduction in household consumption among middle-aged and older adults, it is unlikely to result in a sharp or abrupt decline. Based on this theoretical foundation, the following hypothesis is proposed:

*Hypothesis 1*: Widowhood reduces household consumption among middle-aged and older adults, but the magnitude of the decline is limited.

Rural middle-aged and older households face greater economic shocks following widowhood. Widowhood is often accompanied by substantial losses in income and wealth, and widows tend to be poorer than their married counterparts (18). Empirical studies from China also suggest that rural widows are in a more vulnerable economic position. After spousal income is lost, it is often difficult for them to receive compensatory support from their children, leading to a sharper decline in living standards compared to urban widows (19). Similarly, evidence from Vietnam shows that the probability of falling into poverty increases significantly for widowed households (20). This disparity is especially pronounced in rural areas, where social security systems are weaker and agricultural income is less stable. Based on this reasoning, the following hypothesis is proposed:

*Hypothesis 2.1*: The impact of widowhood on household consumption is significantly greater among rural middle-aged and older adults than among their urban counterparts.

Weir et al. (21), using data from the U. S. Health and Retirement Study (HRS), found that individuals who experienced widowhood before retirement age faced significantly higher poverty risks compared to those widowed after retirement. This suggests that older widowed individuals may smooth income losses and reduce labor through access to social security, whereas middle-aged individuals lack such compensatory mechanisms and must rely on increased labor supply or draw down savings to maintain consumption. Li et al. (22), drawing on data from the CHARLS, provide evidence that more generous pension benefits significantly buffer the detrimental health consequences of widowhood among older adults. Therefore, for the middle-aged, limited coverage by pensions and survivors’ benefits often results in a sharper decline in consumption or living standards (23). In terms of assets and savings, middle-aged widowed individuals also suffer more pronounced losses. Their current consumption drops more steeply, and their future retirement consumption capacity is likewise impaired. Based on this reasoning, the following hypothesis is proposed:

*Hypothesis 2.2*: The consumption impact of widowhood is greater for middle-aged individuals than for older adults.

Widowhood is frequently preceded by a spouse’s serious illness or end-of-life care, which often entails substantial medical expenses. According to Goda et al. (24), the average monthly out-of-pocket medical spending of widowed individuals is approximately 29% higher than that of married individuals in the same period, with this increase largely driven by long-term care costs such as nursing homes. Similarly, McGarry and Schoeni (25) reported that average out-of-pocket medical expenses in the year prior to a spouse’s death reached nearly $5,684—an amount that constituted a considerable share of household income and significantly undermined the financial position of the surviving spouse. Fan and Zick (18) further found that household expenditures on medical care and funeral services surge sharply in the period leading up to widowhood, substantially crowding out other forms of consumption. In contrast, non-medical expenditures such as leisure and durable goods are more flexible and thus more likely to be delayed or reduced. Based on this, the following hypothesis is proposed:

*Hypothesis 2.3*: The impact of widowhood on medical consumption is significantly greater than its impact on non-medical consumption.

Bonnet et al. (26), using data from the European Household Panel on Housing Economics, found that widowed households often downsize their living space or exit homeownership altogether due to a sudden drop in income, resulting in a sharp contraction of housing consumption. Similarly, Hungerford (5), drawing on the U. S. Panel Study of Income Dynamics and the German Socioeconomic Panel, identified the loss of pension income as the primary driver of widow poverty, with reductions in income directly translating into lower overall consumption. Burke et al. (27) document emotional compensation behaviors following spousal loss. Tseng et al. (28) find that although emotional responses among older adults may gradually lessen over time after the death of a spouse, the bereavement period is not merely temporary and significantly contributes to the onset of depression. Furthermore, Nicole and Zachary (29) found that following widowhood, middle-aged households experience a much steeper decline in disposable income compared to older households, leading to a more pronounced contraction in total household spending. Based on this evidence, the following hypothesis is proposed:

*Hypothesis 3.1*: Widowhood significantly reduces total household consumption by lowering household income.

Widowhood brings not only financial hardship but also significant psychological distress. Participation in social clubs and volunteer activities has been shown to effectively buffer the emotional trauma associated with widowhood, and increased spending on leisure and social engagement represents a key pathway for widowed individuals to reconstruct their social roles (30). Using data from the Americans’ Changing Lives study, Janke et al. (31) found that after spousal loss, expenditures on cultural activities, travel, and charitable donations increased by an average of 15%. Furthermore, Glass (32), writing in the field of social science and medicine, confirmed that spending on psychological and emotional well-being is a critical coping mechanism for alleviating loneliness and depressive symptoms among older widowed individuals. Based on this evidence, the following hypothesis is proposed:

*Hypothesis 3.2*: Psychological distress following widowhood leads surviving spouses to increase consumption related to emotional and social well-being.

The death of a spouse significantly increases survivors’ perceived uncertainty about the future, including concerns over reduced future income and health-related (longevity) risks. Kenen (33) finds that widowed individuals experience both objective uncertainty—such as changes in health and financial status—and subjective uncertainty. According to the life-cycle consumption model, when households face income or longevity risks, they tend to increase savings and reduce consumption, particularly non-essential consumption, as a form of precautionary behavior (34). Higher perceived risks related to income or health lead risk-averse households to lower current consumption and raise precautionary savings (35). In this context, widowhood amplifies concerns over future uncertainty and strengthens the precautionary saving motive within households. Based on this reasoning, the following hypothesis is proposed:

*Hypothesis 3.3*: Widowhood increases perceived future uncertainty and induces households to raise their precautionary saving rate.

### 2.2 Data and descriptive statistics

This paper utilizes data from the CHARLS, covering survey waves conducted in 2011, 2013, 2015, 2018, and 2020. CHARLS is a nationally representative longitudinal survey targeting Chinese adults aged 45 and above. It employs multi-stage probability sampling and collects detailed information on demographic and socioeconomic characteristics, household structure, employment status, self-rated health, insurance coverage, financial conditions, and mental health (e.g., depression scores). The survey covers more than 10,000 households across 150 counties and 450 villages or urban neighborhoods in 28 provinces (including municipalities and autonomous regions). To address outliers associated with extremely older adults, the analytical sample is restricted to respondents aged 45 to 95. After data cleaning, a total of 9,890 valid observations were retained.

Widowhood has a direct impact on household income and may further influence consumption decisions. In this paper, household consumption as reported in CHARLS is used as the dependent variable. It includes essential expenditures, discretionary or psychological consumption (e.g., leisure and tourism), medical consumption, and other expenditures such as tobacco, alcohol, and personal care. The key explanatory variable is the “widowhood shock.” Since individual and household characteristics also affect consumption and health outcomes, a set of covariates is included: age, gender, hukou status (household registration), education level, retirement status, household expenditure, household cash and savings, and outstanding household debt (9). Variable definitions and descriptive statistics are presented in Table 1.

**Table 1:** Variable names and definitions.

Variable name	Symbol	Definition
Dependent variables		
Total consumption	*Exp*	Sum of all household expenditures.
Medical consumption	*Med_Exp*	Household spending on medical treatment and healthcare services.
Non-medical consumption	*Non_Med_Exp*	Total consumption minus medical consumption.
Key explanatory variable		
Widowhood shock	*DID*	A policy dummy variable equal to 1 if the respondent experienced spousal loss in 2015, 0 otherwise.
Control variables		
Retirement status	*PR*	Dummy variable: 1 = retired, 0 = not retired.
Age	*Age*	Respondent’s age, restricted to the 45–95 range.
Gender	*Gender*	Dummy variable: 1 = male, 0 = female.
Hukou status	*Hukou*	Household registration type: 1 = urban, 0 = rural.
Education level	*Education*	0 = No formal education, 1 = Preschool, 2 = Primary, 3 = Junior High, 4 = High School, 5 = Vocational, 6 = Associate Degree, 7 = Bachelor, 8 = Master’s or above.
Cash and savings	*Asset*	Total cash and savings held by the household (in RMB).
Outstanding debt	*Loan*	Total unpaid household loans (in RMB).
Depression score	*SRDS*	Depression score; higher values indicate better mental health.
Self-rated health	*SRH*	Self-evaluated health score; higher values indicate better health.
Physical health index	*PH*	Index based on chronic disease composition from physical examination data.
Mechanism variables		
Wage income	*Wage_income*	Household wage income (in RMB).
Psychological consumption	*Mental_Exp*	Spending on leisure, tourism, education/training, and charitable donations.
Saving rate	*Save_ratio*	1 minus the ratio of total consumption to household net assets.

### 2.3 DID model

Although widowhood shock is largely exogenous in nature, this paper nonetheless considers the potential issue of endogeneity from the perspective of omitted variable bias^2^. A key concern is that unobserved common factors shared among family members, such as genetic traits, dietary habits, and health-related behaviors, may influence not only the consumption preferences of the deceased but also those of surviving household members. Some of these omitted factors may be time-invariant, while others may vary over time. If these influences are not properly controlled for, model estimates may suffer from omitted variable bias, potentially leading to endogeneity. To address this issue, a DID estimation strategy is employed, incorporating both individual fixed effects and time fixed effects. Specifically, the DID approach is used to estimate the coefficient φ in Equation 1, based on five waves of CHARLS panel data from 2011 to 2020. This allows us to identify the causal effect of the 2015 widowhood shock on household consumption among middle-aged and older adults.

Regarding the construction of the interaction term in the DID model, this paper defines the control group as middle-aged and older individuals who did not experience widowhood at any point between 2011 and 2020. The treatment group consists of individuals who were not widowed during 2011–2013, experienced widowhood in 2015, and did not experience any further widowhood shock in the 2018–2020 period. The year 2015 is treated as the policy intervention point: years prior to 2015 are defined as the pre-treatment period, and 2015 and subsequent years are defined as the post-treatment period. Based on this structure, an interaction term is constructed to capture the policy variable representing the widowhood shock.

Accordingly, the DID model is specified as follows:


(1) $$\mathrm{Ex}p_{i,t}=\alpha_{0}+{\mathrm{\varphi T}}_{i}{\text{Post}}_{t}+{\mathrm{\lambda T}}_{i}+{\text{μPost}}_{t}+\beta X_{i,t}+I_{i}+S_{t}+\varepsilon_{i,t}$$

In Equation 1, Expit represents the consumption level of individual i in year *t*. α0 is the constant term. Ti is a dummy variable indicating group assignment (treatment group = 1, control group = 0). Postt is a policy shock variable indicating the timing of widowhood, equal to 1 if the individual became widowed in 2015, and 0 otherwise. Xit denotes the vector of control variables. Ii captures individual fixed effects, and Si denotes year fixed effects, which are included to account for the influence of macro-level shocks on household consumption. εit is the error term.

Table 2 reports descriptive statistics for the treatment and control groups. As shown in the table, household consumption is significantly lower among widowed individuals compared to their non-widowed counterparts. Both income and psychological consumption are also lower in the treatment group. Notably, the saving rate is higher among widowed individuals, indicating a contraction in relative consumption. In some cases, the saving rate is negative, reflecting the fact that, due to factors such as mortgage payments and other household debt, household consumption can exceed household net assets in recent years in China.

**Table 2:** Descriptive statistics.

Variable		Widowed (treatment group)				Non-widowed (control group)			
		Mean	Median	SD	*N*	Mean	Median	SD	*N*
Dependent variables	Total consumption	6.158	7.606	3.983	498	7.565	8.561	3.258	9,392
	Medical consumption	5.029	6.217	3.755	439	5.266	6.909	3.732	9,032
	Non-medical consumption	5.665	6.910	3.777	498	7.068	7.969	3.143	9,392
Key explanatory variable	Widowhood dummy	0.600	1	0.490	498	0	0	0	9,392
Control variables	Retirement status	0.0984	0	0.298	498	0.103	0	0.304	9,392
	Age	65.78	66.02	9.250	498	60.46	59.36	8.867	9,392
	Gender	0.201	0	0.401	498	0.410	0	0.492	9,392
	Hukou	0.163	0	0.369	498	0.201	0	0.401	9,392
	Education level	1.161	1	1.529	498	1.735	2	1.512	9,392
	Log (cash and savings)	3.587	4.615	3.572	498	4.611	5.707	3.987	9,392
	Log (Debt)	0.255	0	1.607	498	0.491	0	2.225	9,392
	Depression score	6.147	7	2.654	415	7.231	8	2.367	8,067
	Self-rated health	1.777	2	0.935	449	2.045	2	0.935	8,552
	Physical health index	4.353	5	1.535	498	4.517	5	1.483	9,392
Mechanism variables	Wage income	1.003	0	2.890	498	1.858	0	3.832	9,380
	Psychological consumption	2.168	0	3.571	446	2.597	0	3.613	9,036
	Saving rate	−30.73	−0.475	147.7	275	−31.43	0.197	623.5	6,007

## 3 Results

### 3.1 The impact of widowhood on household consumption among middle-aged and older adults

As China undergoes a profound demographic shift toward an aging society, its population structure is gradually evolving into an inverted pyramid. While birth, aging, illness, and death are natural parts of life, the sudden loss of a spouse for middle-aged and older adults often entails not only the collapse of emotional support but also the erosion of economic stability. In order to maintain basic living standards, households may be forced to reduce consumption. Moreover, spousal death also marks the end of a marital relationship. For the surviving partner, the emotional trauma may further influence consumption behavior, particularly among older individuals, whose decisions are shaped not only by economic necessity but also by psychological adjustment.

To assess the robustness of the baseline results, Table 3 presents the estimated effects of widowhood on household consumption under different model specifications. Specifically, the variable DID captures the widowhood shock. Model (1) excludes both time fixed effects and control variables; Model (2) includes time fixed effects but omits control variables; Model (3) includes control variables but not time fixed effects; and Model (4) presents the fully specified model with both time fixed effects and controls^3^. Across all four specifications, the estimated coefficients for DID are consistently negative and statistically significant. On average, household consumption among middle-aged and older adults who experienced widowhood declined by 1.019%. Although this decline appears modest in magnitude, it is consistent with the life-cycle hypothesis. According to this theory, households tend to reduce consumption only marginally when facing adverse shocks in order to smooth consumption and maximize long-term utility. This finding provides strong support for Hypothesis 1. In addition, we observe a clear positive relationship between household assets (Ln_*Asset*) and consumption: households with higher asset levels tend to spend more, which aligns with standard economic intuition.

**Table 3:** The impact of widowhood on household consumption among middle-aged and older adults.

Variables	(1)	(2)	(3)	(4)	(5)
*DID*	−0.827***	−0.827***	−0.947***	−1.019***	
	(0.311)	(0.310)	(0.360)	(0.348)	
*DID_2013*					0.021
					(0.587)
*DID_2015*					−1.362**
					(0.606)
*DID_2018*					−1.419***
					(0.517)
*DID_2020*					0.185
					(0.441)
*Post*	0.649^***^	1.307^***^	−0.520^***^	1.798^***^	
	(0.065)	(0.075)	(0.139)	(0.321)	
*PR*			0.171	0.023	0.032
			(0.249)	(0.240)	(0.241)
*Ln_Age*			9.111^***^	−0.944	−0.832
			(1.261)	(1.928)	(1.928)
*Gender*			0.154	0.045	0.043
			(0.927)	(0.896)	(0.896)
*Hukou*			−0.117	0.095	0.094
			(0.216)	(0.213)	(0.213)
*Education*			0.028	0.043	0.041
			(0.093)	(0.092)	(0.092)
*Ln_Asset*			−0.030^***^	0.087^***^	0.085***
			(0.011)	(0.014)	(0.014)
*Ln_Wage_income*			−0.002	−0.002	−0.002
			(0.014)	(0.013)	(0.013)
*Ln_Loan*			0.005	0.018	0.017
			(0.019)	(0.019)	(0.019)
*SRDS*			−0.043^**^	0.002	0.001
			(0.021)	(0.021)	(0.021)
*SRH*			−0.096^*^	−0.053	−0.054
			(0.052)	(0.051)	(0.051)
*PH*			−0.137^***^	−0.012	−0.010
			(0.042)	(0.043)	(0.043)
Constant	7.127***	7.606***	−28.218***	11.134	10.625***
	(0.039)	(0.060)	(5.220)	(7.745)	(7.885)
Time fixed effects	NO	YES	NO	YES	YES
Individual fixed effects	YES	YES	YES	YES	YES
*N*	9,890	9,890	8,253	8,253	8,190
*R* ^2^	0.012	0.086	0.032	0.083	0.174

***p < 0.01, **p < 0.05, *p < 0.10. Robust standard errors are reported in parentheses.

### 3.2 Robust test

#### 3.2.1 Parallel trends and placebo tests

**Figure 1:**
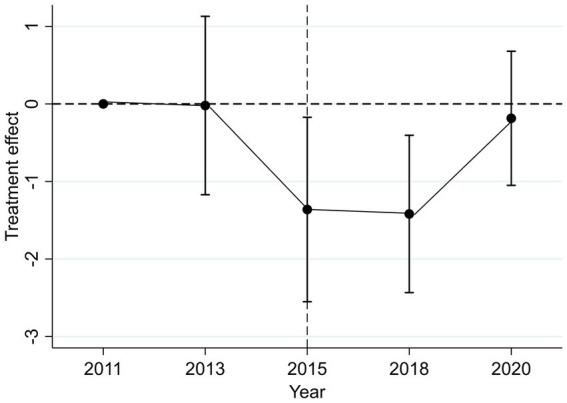
Dynamic effects of widowhood on household consumption.

Although Table 3 has already reported the DID estimation results, the validity of these estimates hinges on the key assumption that the treatment and control groups exhibit parallel trends in consumption prior to the widowhood shock. In other words, the two groups should display similar pre-treatment trends in the outcome variable. In addition, this paper aims to examine the dynamic effects of widowhood over time. To this end, an event study approach is employed to estimate the temporal pattern of the treatment effect. The corresponding results are shown in Figure 1 and Model (5) of Table 3. The event study estimates (Figure 1) indicate that there is no statistically significant difference in consumption between the treatment and control groups during the two pre-treatment periods (2011 and 2013). This finding provides empirical support for the validity of the parallel trends assumption. Notably, the year 2011 is used as the baseline period. Furthermore, Model (5) in Table 3 demonstrates that even when 2013 is artificially treated as a placebo intervention point, the results remain statistically insignificant, offering additional confirmation that the parallel trends assumption holds. On the other hand, a significant decline in household consumption is observed in the periods following the widowhood shock. However, this adverse effect appears to diminish over time. In the short run, the shock leads to a sharp drop in consumption, but as time progresses, consumption gradually recovers. In other words, in the absence of further external interventions, household consumption tends to revert to its pre-shock level over time. This suggests that the negative impact of widowhood may be partially mitigated through an adaptive or self-correcting adjustment process.

**Figure 2:**
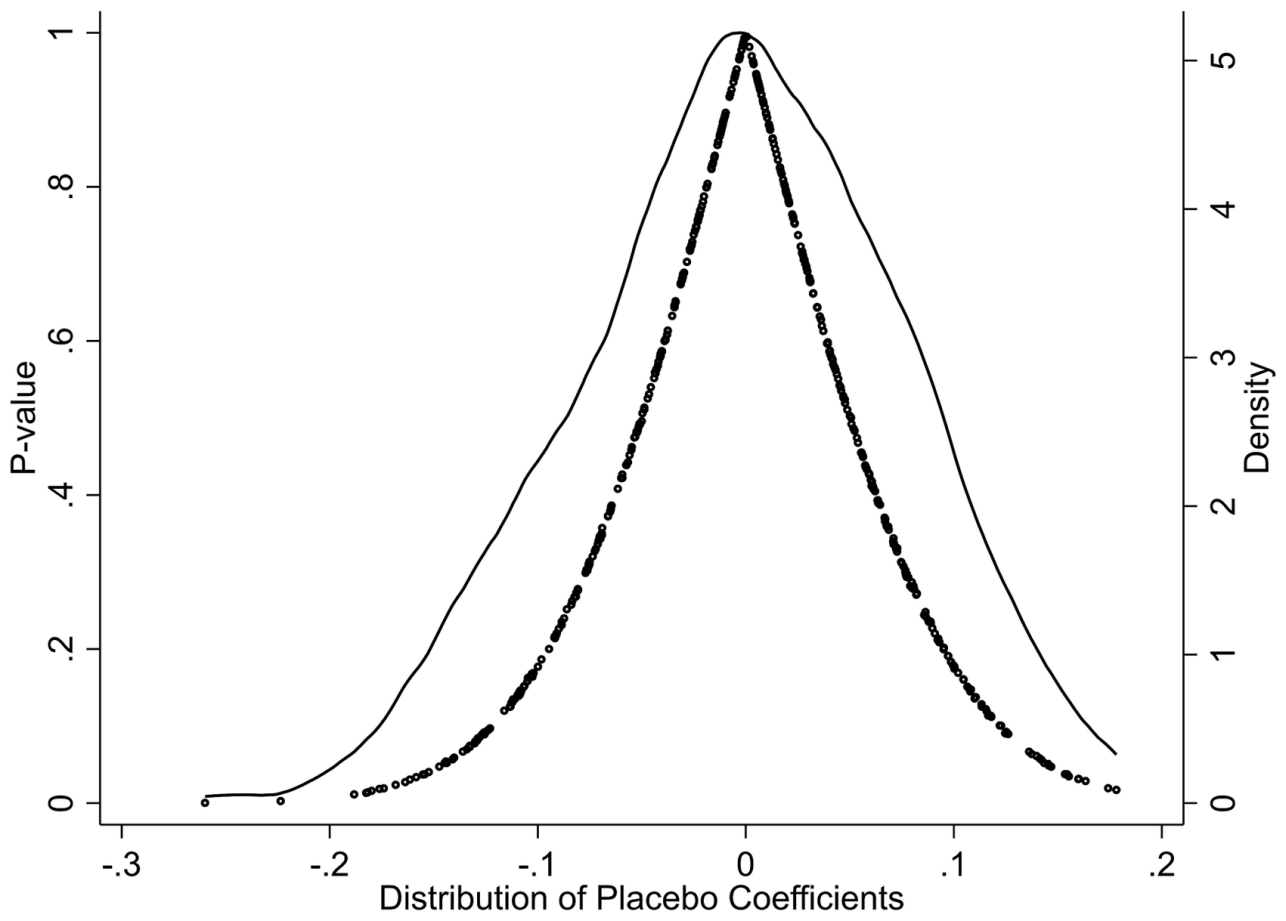
Placebo test results based on 500 random permutations.

In the placebo test shown in Figure 2, 500 randomly generated “pseudo-DID” coefficients and their corresponding *p*-values are plotted. The results indicate that nearly all of the placebo estimates are centered around zero on the horizontal axis, with associated p-values above 0.05. The kernel density curve exhibits a sharp peak near zero, suggesting no systematic or significant “false effect” is produced when the treatment group indicator is randomly reassigned. This finding provides strong evidence that the observed treatment effect in the study is neither an artifact of parallel trends nor the result of random noise. The placebo test is fully passed, thereby reinforcing the causal validity of the impact of widowhood on household consumption among middle-aged and older adults.

#### 3.2.2 Subsample regression

Although the parallel trends assumption has been tested from multiple perspectives, it is still necessary to further assess the robustness of the results. To begin with, some older individuals in the sample are well above the average age. Since their consumption may depend more on their children than on their spouse, the sample is restricted to individuals aged between 45 and 85 in order to address potential bias related to extreme age values [Model (1)]. In addition, households with extremely high levels of consumption tend to be wealthier, and may therefore be less affected by the loss of a spouse. To test this possibility, we exclude the top 1 % of the sample in terms of total consumption [Model (2)]. Moreover, due to the global outbreak of COVID-19 at the end of 2019, some widowhood cases in 2020 may have been directly caused by the pandemic. To control for this factor, all observations from the year 2020 are excluded from the sample [Model (3)]. It is widely recognized that men and women may differ in their approaches to consumption. Whether such gender differences persist under the specific condition of widowhood warrants further investigation. To explore this, we estimate the impact of widowhood on male consumption separately [Model (4)], and on female consumption [Model (5)]. The results reported in Table 4 show that all estimated coefficients are significantly negative. There is no meaningful difference between men and women in terms of the change in consumption following widowhood. These findings confirm the robustness of the main results across different model specifications and sample adjustments.

**Table 4:** Robust test: based on subsample regressions.

Variables	(1)	(2)	(3)	(4)	(5)
*DID*	−1.063***	−0.932***	−1.358***	−0.957*	−0.990**
	(0.348)	(0.356)	(0.390)	(0.560)	(0.416)
*Post*	1.811***	1.778***	−0.039	1.532***	1.956***
	(0.330)	(0.322)	(0.290)	(0.515)	(0.408)
*PR*	0.023	0.009	−0.036	0.155	−0.129
	(0.240)	(0.242)	(0.339)	(0.331)	(0.353)
*Ln_Age*	−1.099	−0.875	−0.771	1.050	−2.265
	(1.980)	(1.934)	(2.291)	(3.024)	(2.465)
*Gender*	0.045	−0.111	−0.332	−0.188	0.263
	(0.896)	(0.891)	(0.948)	(0.315)	(0.284)
*Hukou*	0.083	0.112	0.157	0.072	0.038
	(0.213)	(0.210)	(0.274)	(0.137)	(0.124)
*Education*	0.039	0.075	0.086	0.084^***^	0.090^***^
	(0.093)	(0.091)	(0.114)	(0.023)	(0.018)
*Ln_Asset*	0.085^***^	0.091^***^	0.092^***^	−0.003	−0.003
	(0.014)	(0.014)	(0.016)	(0.018)	(0.020)
*Ln_Wage_income*	−0.002	−0.001	0.006	0.037	0.003
	(0.013)	(0.014)	(0.017)	(0.026)	(0.028)
*Ln_Loan*	0.018	0.017	0.010	0.014	−0.009
	(0.019)	(0.019)	(0.022)	(0.039)	(0.024)
*SRDS*	0.000	0.002	0.010	0.037	−0.097
	(0.021)	(0.021)	(0.025)	(0.090)	(0.061)
*SRH*	−0.055	−0.062	−0.006	−0.124	0.026
	(0.051)	(0.051)	(0.060)	(0.087)	(0.050)
*PH*	−0.015	−0.016	−0.064	0.155	−0.129
	(0.043)	(0.043)	(0.061)	(0.331)	(0.353)
Constant	11.799	10.837	10.550	3.332	16.494*
	(7.942)	(7.773)	(9.214)	(12.134)	(9.934)
Time fixed effects	YES	YES	YES	YES	YES
Individual fixed effects	YES	YES	YES	YES	YES
*N*	8,224	8,163	6,731	3,231	5,022
*R* ^2^	0.083	0.084	0.037	0.094	0.076

***p < 0.01, **p < 0.05, *p < 0.10. Robust standard errors are reported in parentheses.

### 3.3 Heterogeneity analysis

The household head is typically the primary decision-maker in family financial matters, and it is therefore important to examine how characteristics of the household head influence consumption under the shock of widowhood. In China, the hukou system creates a structural divide between urban and rural residents, which in turn affects access to social security and public services. Age is also closely linked to life experience and psychological resilience. These individual characteristics of the household head may lead to heterogeneous effects on consumption. Moreover, widowhood has a direct impact on psychological well-being, which may trigger medical-seeking behavior and alter both medical and non-medical consumption patterns. In light of this, three dimensions of heterogeneity are examined: (1) differences in consumption based on hukou status (urban = 1, rural = 0); (2) age-based differences, using 60 years as the cutoff to distinguish middle-aged (under 60) from older adults (60 and above); and (3) differences in medical consumption (ln_*Eexpense*) versus non-medical consumption (ln_*nonEexpense*). Subsample regressions are conducted to explore the heterogeneity of the treatment effect across these groups. The results are presented in Table 5.

**Table 5:** The impact of individual characteristics on household consumption among middle-aged and older adults.

Variables	ln*_Exp*				ln*_Eexpense*	ln_*nonEexpense*
	Urban (1)	Rural (2)	Age ≥ 60 (3)	Age < 60 (4)	Medical consumption (5)	Non-medical consumption (6)
*DID*	−1.386	−0.889**	−1.126**	−1.422**	−1.014***	−0.791**
	(1.245)	(0.364)	(0.492)	(0.670)	(0.341)	(0.323)
*Post*	2.693***	1.537***	1.452**	2.878***	5.657***	0.821***
	(0.735)	(0.380)	(0.595)	(0.699)	(0.393)	(0.313)
*Gender*	6.197^***^	−0.673	1.083	−0.220	1.061	−0.362
	(2.028)	(0.839)	(1.279)	(1.080)	(0.963)	(0.882)
*PR*	−0.463	0.411	0.148	0.080	−0.116	0.175
	(0.318)	(0.407)	(0.410)	(0.422)	(0.277)	(0.238)
*Ln_Age*	−6.179	0.548	1.304	−6.404	1.649	0.745
	(4.103)	(2.311)	(4.218)	(4.219)	(2.385)	(1.883)
*Hukou*			0.395	−0.143	−0.048	0.091
			(0.359)	(0.291)	(0.196)	(0.200)
*Education*	−0.077	0.089	0.035	0.001	0.163^*^	−0.013
	(0.170)	(0.113)	(0.152)	(0.149)	(0.092)	(0.092)
*Ln_Asset*	0.033	0.091^***^	0.082^***^	0.081^***^	0.036^***^	0.080^***^
	(0.032)	(0.016)	(0.022)	(0.020)	(0.013)	(0.013)
*Ln_Wage_income*	−0.062^*^	0.009	−0.027	0.012	0.015	0.004
	(0.034)	(0.015)	(0.028)	(0.017)	(0.012)	(0.013)
*Ln_Loan*	0.003	0.020	0.031	0.008	−0.002	0.018
	(0.049)	(0.021)	(0.049)	(0.023)	(0.016)	(0.019)
*SRDS*	−0.022	0.013	−0.046	0.040	0.021	−0.002
	(0.060)	(0.023)	(0.031)	(0.029)	(0.019)	(0.020)
*SRH*	−0.079	−0.040	−0.028	−0.067	−0.256^***^	0.006
	(0.147)	(0.055)	(0.080)	(0.073)	(0.052)	(0.049)
*PH*	−0.023	0.017	−0.002	0.390	−0.043	0.046
	(0.113)	(0.046)	(0.051)	(0.584)	(0.041)	(0.041)
Constants	30.757*	5.052	1.653	30.533*	−6.188	4.269
	(16.582)	(9.283)	(17.428)	(17.758)	(9.581)	(7.569)
Time fixed effects	Yes	Yes	Yes	Yes	Yes	Yes
Individual fixed effects	Yes	Yes	Yes	Yes	Yes	Yes
*N*	1,638	6,615	3,907	4,346	7,982	8,253
*R* ^2^	0.072	0.088	0.093	0.062	0.481	0.086

***p < 0.01, **p < 0.05, *p < 0.10. Robust standard errors are reported in parentheses.

As shown in Table 5, the impact of widowhood on consumption varies significantly across subgroups. Among urban middle-aged and older adults [Model (1)], there is no statistically significant decline in consumption following widowhood. In contrast, a significant reduction is observed among rural residents in the same age group [Model (2)]. This disparity can be attributed to two main factors: first, the urban social security system is generally more comprehensive; second, urban households tend to have higher overall income levels. As a result, urban families are better equipped to absorb the negative economic shock caused by widowhood. When comparing across age groups, consumption declines significantly for both middle-aged and older individuals. However, the magnitude of the decline is greater among the middle-aged [Model (4)] than among the older adults [Model (3)]. This may be because middle-aged individuals are typically the primary income earners in the household, and the loss of their labor significantly reduces household income. In contrast, older adults are more likely to rely on fixed retirement benefits and support from their children, making the relative impact of widowhood on income less severe. In terms of consumption structure, the decline in medical consumption after widowhood [Model (5)] is greater than the decline in non-medical consumption [Model (6)]. One explanation is that significant medical expenses are often incurred prior to the spouse’s death, resulting in a post-shock decrease in such expenditures. At the same time, the reduction in household income following widowhood also constrains both medical and non-medical consumption. These findings provide empirical support for Hypotheses 2.1 through 2.3.

## 4 Discussion

### 4.1 Potential mechanisms of impact

Although this paper has confirmed that widowhood has a significant negative impact on household consumption, the underlying transmission mechanisms remain unclear. We therefore explore the possible mechanisms of influence from both direct and indirect perspectives. From the standpoint of direct effects, widowhood reduces total household income and may even push households into poverty—a finding supported by prior studies (3–6). In addition, widowhood has been shown to impair the psychological well-being of surviving household members (1, 2). To cope with such emotional trauma, households may increase spending on psychological or emotional needs, such as leisure, entertainment, or social participation. On the indirect side, the income reduction caused by widowhood may lead households to increase precautionary savings, as predicted by the life-cycle theory. In this case, the household saving rate is likely to rise as a form of financial self-protection. Based on this framework, we examine three potential mechanisms through which widowhood may affect consumption: income loss, increased psychological consumption, and a higher household saving rate.

To test the income mechanism, this paper uses annual household income (ln*Wage_income*) as a measure of economic resources. As shown in Model (1) of Table 6, household income declines significantly following the widowhood shock. In response to this temporary hardship, households tend to reduce their consumption to maintain financial stability. To test the psychological consumption mechanism, the sum of expenditures on recreation, tourism, educational training, and charitable donations is used as the measure of psychological consumption (ln*Mental_Exp*). As indicated in Model (2) of Table 6, surviving household members significantly increased psychological consumption, likely as a way to cope with emotional distress widowhood. Finally, to test for changes in precautionary savings, the saving rate is measured as one minus the ratio of total consumption to household net assets (*Save_ratio*). According to Model (3) in Table 6, the saving rate rises significantly by 46.953% after the widowhood shock. This indicates that households responded to future uncertainty by substantially raising their precautionary savings. These findings provide strong empirical support for Hypotheses 3.1 through 3.3.

**Table 6:** Regression results for mechanism analysis.

Variables	(1) ln*Wage_income*	(2) ln*Mental_Exp*	(3) *Save_ratio*
*DID*	−0.756**	0.762**	46.953***
	(0.330)	(0.312)	(16.859)
*Post*	0.364	1.622***	−33.520
	(0.324)	(0.331)	(20.992)
*PR*	−1.672^***^	−0.262	8.281
	(0.398)	(0.303)	(7.356)
*Ln_Age*	8.341^***^	5.404^***^	56.324
	(2.038)	(1.973)	(133.444)
*Gender*	−0.889	1.649^*^	9.011
	(0.956)	(0.867)	(9.550)
*Hukou*	0.056	0.090	−0.418
	(0.211)	(0.233)	(8.791)
*Education*	−0.023	0.098	0.777
	(0.122)	(0.110)	(5.531)
*Ln_Asset*	0.028^**^	0.025^*^	17.216^***^
	(0.013)	(0.014)	(4.999)
*Ln_Wage_income*		0.026^*^	3.468^***^
		(0.014)	(0.744)
*Ln_Loan*	0.017	0.001	4.709^***^
	(0.020)	(0.021)	(0.717)
*SRDS*	0.103^***^	−0.008	3.705
	(0.019)	(0.022)	(5.485)
*SRH*	0.068	0.037	−4.820
	(0.051)	(0.057)	(7.030)
*PH*	0.166^***^	0.157^***^	10.746
	(0.035)	(0.040)	(11.272)
Constant	−32.738***	−22.105***	−443.505
	(8.208)	(7.956)	(527.855)
Time fixed effects	YES	YES	YES
Individual fixed effects	YES	YES	YES
*N*	8,253	7,967	5,694
*R* ^2^	0.048	0.071	0.013

***p < 0.01, **p < 0.05, *p < 0.10. Robust standard errors are reported in parentheses.

### 4.2 Directions for future research

With the deepening of population aging in China, the country has gradually formed a “9,073” older adults care structure, in which approximately 90 percent of older adults receive care at home, around 7 percent rely on community-based care, and only 3 percent reside in institutional facilities. Under this system, addressing the physical and mental well-being of the older adults, as well as their financial pressures, has become an urgent policy priority. In 2022, the State Council of China issued the “14th Five-Year Plan for the Development of the National Aging Undertaking and Older Adults Care Service System.” This plan outlines development goals for the period, including the expansion of older adults care services, improvement of the health support system for older adults, integrated innovation across diverse service models, enhanced resource guarantees, and the creation of a more age-friendly living environment. The plan further aims to foster a nationwide consensus and institutional framework for actively responding to population aging, thereby significantly improving the sense of fulfillment, happiness, and security among the older adults. For older individuals, both emotional and economic needs deserve close attention. The findings of this paper contribute to the broader understanding of these dual challenges and offer policy-relevant insights for advancing China’s aging agenda. Moreover, population aging is also a pressing issue in developed economies such as those in Europe and North America. Whether widowhood occurs in midlife or in later life, the conclusions drawn from this paper offer valuable implications with broader international relevance.

Nevertheless, although this paper investigates the impact of widowhood on household consumption among middle-aged and older adults and employs a DID approach to address potential bidirectional causality, further improvements could be made by identifying exogenous instrumental variables. The use of such instruments would allow for stronger causal inference and provide additional robustness to the findings. In addition, other economic outcomes associated with widowhood remain worth exploring. For instance, the effect of widowhood on household asset allocation, particularly changes in the composition of financial assets, deserves further investigation. Since widowhood is one specific form of bereavement, a broader discussion encompassing other types of bereavement and a comparative analysis of different bereavement scenarios could enhance our understanding of the broader risks associated with loss. Despite these limitations, this paper contributes meaningful empirical evidence from China to the field of health economics.

## 5 Conclusion

This paper uses data from the CHARLS from 2011 to 2020 and applies a DID approach to examine the impact of widowhood on household consumption among middle-aged and older adults, as well as the mechanisms through which this effect operates. The results show that widowhood leads to a reduction in household consumption. However, the magnitude of the decline is relatively modest. This finding is consistent with the life-cycle hypothesis, which suggests that households tend to smooth consumption over time to maximize long-term utility and avoid large fluctuations. Heterogeneity analysis reveals that the adverse effect of widowhood is more pronounced among rural households, likely due to their lower income levels and weaker social security systems. In contrast, the effect is not statistically significant for urban households. The impact is also stronger among middle-aged individuals than among older adults, and the reduction in medical consumption is greater than that in non-medical consumption. Mechanism analysis indicates that widowhood directly reduces household consumption by lowering income and increasing psychological consumption. Indirectly, it leads to higher saving rates, which further suppress consumption.

Based on the findings of this paper, the following policy recommendations are proposed: First, improve social security and psychological support systems. The government should strengthen both the financial protection and emotional care provided to widowed middle-aged and older individuals. Enhancements to pension systems and social assistance programs are needed, including the introduction of survivor benefits or living subsidies specifically for the widowed older adults. These measures would help stabilize their consumption and standard of living widowhood. In parallel, community-based psychological support networks should be developed, offering grief counseling services and peer support groups. A combination of financial assistance and mental health support can help mitigate the negative effects of widowhood on consumption confidence and quality of life.

Second, strengthen income and healthcare protection for rural middle-aged and older individuals in a context-specific manner. Given that widowhood has a greater negative impact on consumption in rural and middle-aged populations, targeted efforts in these areas are essential. The government should increase the benefits under the basic pension system for rural and urban residents, improve rural minimum subsistence allowances and temporary assistance schemes, and prevent widowed individuals from falling into poverty due to sudden income loss. Furthermore, the rural healthcare system should be improved by raising the reimbursement rates of the Basic Medical Insurance for Urban and Rural Residents and Catastrophic Illness Insurance, ensuring that widowed individuals can afford necessary healthcare. Local governments are encouraged to explore rural mutual aid models and mobilize village collectives and community organizations to provide daily care and health monitoring services for older adults living alone after widowhood.

Third, promote household-level financial literacy and preparedness for widowhood-related risks. At the individual and household levels, there is a need to strengthen public awareness of risk management and long-term financial planning. Governments and communities should conduct financial education programs that emphasize the importance of saving and insurance over the life course. Households should be encouraged to build precautionary savings and purchase appropriate insurance products, such as pension and life insurance, to protect surviving spouses. While precautionary saving is important, it should be balanced to avoid adversely affecting current living standards. Establishing emergency funds and financial buffers can help households better maintain stability in consumption and well-being when facing widowhood.

## Data Availability

Publicly available datasets were analyzed in this study. This data can be found: http://charls.pku.edu.cn/.

## Footnotes

^1^The data is sourced from: https://www.stats.gov.cn/.

^2^Endogeneity concerns in this paper primarily arise from two sources: omitted variable bias and reverse causality. Regarding the latter, Jones and Olken (36) provide empirical evidence that death is a relatively exogenous event. Their findings suggest that, aside from age and gender, most individual characteristics have little predictive power over mortality, implying that reverse causality is unlikely to pose a serious endogeneity threat. Therefore, the use of the DID method in this paper helps effectively address the issue of reverse causality.

^3^Model (5) reports the results from the event study approach, which will be discussed in detail in a later section.
